# One step forward, one step sideways? Expanding research capacity for neglected diseases

**DOI:** 10.1186/1472-698X-10-20

**Published:** 2010-07-14

**Authors:** Joel Lexchin

**Affiliations:** 1 School of Health Policy and Management, York University, 4700 Keele St., Toronto ON M3J 1P3, Canada; 2 Emergency Department, University Health Network, 190 Elizabeth St., Toronto ON M5G 2C4, Canada; 3 Department of Family and Community Medicine, University of Toronto, 263 McCaul St., Toronto ON M5T 1W7, Canada

## Abstract

**Background:**

There is general agreement, including from the pharmaceutical industry, that current market based methods of generating research into the development of pharmaceutical products that are relevant for developing countries do not work. This conclusion is relevant not just for the most neglected diseases such as leishmaniasis but even for global diseases such as cancer and cardiovascular disease.

**Discussion:**

Stimulating research will mean overcoming barriers such as patent thickets, poor coordination of research activities, exclusive licensing of new technologies by universities and the structural problems that inhibit conducting appropriate clinical trials in developing countries. In addition, it is necessary to ensure that the priorities for research reflect the needs of developing countries and not just donors. This article will explore each of these issues and then look at three emerging approaches to stimulating research -paying for innovation, priority review sales or vouchers and public-private partnerships, - and evaluate their strengths and weaknesses.

**Summary:**

All of the stakeholders agree that there is a pressing need for a major expansion in the level of R&D. Whatever that new model turns out to be, it will have to deal with the 5 barriers outlined in this paper. Finally, none of the three proposals considered here for expanding research is free from major limitations.

## Background

Research and development (R&D) of new medicines is driven by the market where the market typically means large numbers of potential patients with chronic diseases who live in first world countries. These people either have the ability to purchase medicines on their own or, as is more often the case, the cost of medicines is covered publicly. Under these circumstances, companies are able to realize sustained substantial rates of return [1] and are willing to invest in the necessary R&D.

There is general agreement that this model for R&D has failed when it comes to neglected diseases. This assessment comes not just from critics of pharmaceutical companies but from within the industry itself. Speaking to a reporter from the Financial Times, Daniel Vasella, the CEO of Novartis, said "We have no model which would (meet) the need for new drugs in a sustainable way ... You can't expect for-profit organization[s] to do this on a large scale. If you want to establish a system where companies systematically invest in this kind of area, you need a different system." [2]

Evidence of market failure is not hard to document. Between 1975 and 2004 only 21 out of 1556 marketed new chemical entities were indicated for neglected diseases [3]. In spring 2001, the 20 top-grossing pharmaceutical companies in the world were surveyed about recent drug development activity for 5 neglected diseases - Chagas disease, leishmaniasis, malaria, sleeping sickness and tuberculosis. Eleven companies responded; 8 had spent nothing on Chagas disease, leishmaniasis and sleeping sickness and 7 spent less than 1% of their total R&D budget on any of the 5 diseases [4]. In the ensuing years little changed with respect to industry initiated R&D for neglected diseases; 5 out of 12 of the top multinational companies were not conducting any research and these companies were unwilling to enter this area regardless of any incentives offered to them [5]. Furthermore, many of the (few) drugs that industry had developed were of low overall value to developing countries because they were poorly suited to situations in these countries; for example, they needed to be administered within a hospital setting, were not affordable, had poor efficacy or a poor safety profile [5].

The reason behind these failures had little if anything to do with any scientific issues. The World Health Organization considered the scientific feasibility of developing new drugs for 7 major neglected diseases to be high [6]. "Key factors explaining the poor health performance of industry-developed drugs are: industry R&D choices based on primary Western priorities (safety and efficacy); insufficient focus on additional developing country issues such as suitability and likely end price; pressure on companies to balance the cost-benefit equation by maximising the Western market for their products (eg focusing on Western strains and needs); lack of company knowledge; lack of public input; and the need for companies to limit risk and liability, for instance, by excluding paediatric patients and pregnant women." [5]

Although most of the focus has been on drugs for very neglected diseases such as leishmaniasis and Chagas disease it needs to be recognized that the problem in researching drugs for diseases in developing countries is much wider. Conditions such as Chagas disease are usually referred to as Type III diseases, ones that are overwhelmingly or exclusively incident in developing countries, and since there is no developed country demand there is no R&D taking place. (See Table 1.) Type I diseases are incident in both rich and poor countries, with large numbers of vulnerable populations in each and include both communicable diseases e.g., measles and noncommunicable diseases, e.g., diabetes, and tobacco-related illnesses. Type II diseases are incident in both rich and poor countries, but with a substantial proportion of the cases in the poor countries, e.g., HIV/AIDS and tuberculosis. Although Types I and II attract pharmaceutical company R&D activity, drugs developed for them may not be appropriate for developing countries. In Type I diseases there is a lack of incentive to invest in the search for preventive, diagnostic and curative interventions adapted to the resources and social and economic conditions of developing countries while in Type II diseases the type or strain of the disease in developing countries frequently differs from that in developed countries, e.g., strains of HIV differ between developed and developing countries [7].

**Table 1:** Types of diseases

Type	Definition	Example	Barriers to therapy in developing countries
Type 1	Diseases that are incident in both developed and developing countries with large numbers of vulnerable populations in both groups of countries	Diabetes	No market incentive to invest in interventions adapted to the resources and social and economic conditions of developing countries

Type 2	Diseases that are incident in both developed and developing countries but with a substantial proportion of the cases in developing countries	HIV	Type or strain of disease different in developed and developing countries

Type 3	Diseases that are overwhelmingly or exclusively incident in developing countries	Chagas disease	No market incentive to invest in research

While Type III diseases are still very serious they are assuming a less important role in overall population health [8] and aside from HIV/AIDS mortality from communicable diseases is projected to continue decline [9]. Outside of one region in Africa the majority of the disease burden in people over 14 years of age is due to a combination of non-communicable diseases and injury making research into treatments for Type I and II diseases in developing countries imperative.

The next section of this article will explore some of the main systematic barriers to expanding research capacity and look at solutions, using existing examples where possible, to overcoming these barriers. Next, I will analyze three specific proposals that are being advanced for increasing R&D into diseases that are of primary concern to developing countries and look at the strengths and weaknesses of each of these proposals.

## Discussion

### Barriers to expanding research capacity

Barriers to expanding research capacity exist at many levels. There will never be enough money to tackle all of the health problems of the developing world simultaneously and therefore, it is necessary to decide which diseases deserve the most attention. That decision is likely to be made by multiple different actors, which means that there will need to be a measure of research coordination amongst all of the players. In considering pharmaceutical research the issue of intellectual property rights or patents is always front and centre. Universities are often the site of much of the fundamental research behind new medicines but increasingly universities are restricting access to patents that they have taken out on the fruits of their research in the hopes of a large financial windfall from an exclusive license.

Medical research does not arise *de novo *but builds on work that has previously been done. Especially in the case of biotechnology, there may be many steps behind any new research and each of those steps may be patented and those patents held by different entities. Unless there is cooperation in sharing those patents research could be seriously impeded. Finally, once there is a new medicine ready for testing it is essential that clinical trials be conducted on the most relevant populations and in the case of neglected diseases these are populations in developing countries. In order to be able to do trials in these settings the countries in question have to have the capacity to support clinical trials.

#### i. Priority setting

There may be general agreement that new methods need to be found to advance research into diseases affecting developing countries but to date that agreement has not translated into funding that is proportional to the burden of disease in these areas. World Health Organization (WHO) funding in both the Western Pacific and African regions is heavily skewed towards infectious diseases while funding for non-communicable diseases and injury and violence is significantly below the contribution that they make to global mortality and disability-adjusted life years (DALY). This mismatch in funding is even more pronounced in the WHO's extra-budgetary revenue than in its regular budget [10]. Extra-budgetary revenue comes from voluntary contributions mostly from governments, foundations and the United Nations system and are allocated according to donors' preferences, mainly for specific disease-control programs.

Even within the world of communicable diseases donor funding does not correlate with the burden of disease. Shiffman [11] calculated donor funding for 20 historically high-burden communicable diseases for the years 1996 to 2003 from 42 major donors, classifying grants according to the communicable disease targeted. Annual donor dollars per DALY ranged from almost $2500 for polio to $0.58 for acute respiratory infections. According to Shiffman the pattern of donation does not fully reflect either the needs of the recipients in terms of humanitarian concerns and the most pressing problems of people in developing nations nor is it fully explained by a provider perspective, that is, the interests of constituencies in industrialized states. A third factor may be that donors are imitating one another especially, following the lead of large funders such as the Gates Foundation [12].

Imitating the actions of large donors may be especially problematic when one funder dominates the scene as is the case with respect to the Gates Foundation and malaria research. In the late 1990s as little as $84 million annually was being spent but since 2000 the Gates Foundation has put $1.2 billion into the effort. The chief of the malaria program at the WHO alleged that the consequence of the growing dominance of the Gates Foundation was a stifling in the diversity of views among scientists[12]. Others have criticized the Gates Foundation for having a "narrowly conceived understanding of health as the product of technical interventions divorced from economic, social, and political contexts" pointing to the Grand Challenges issued by the Foundation as example of this orientation [13].

The data cited here do not necessarily directly translate into spending on new drug research for diseases in developing countries but it is reasonable to assume that if overall health care funding is heavily skewed then research money will follow the same pattern. This assumption is reinforced by looking at the composition of the leadership of the large health-related public-private partnerships (PPPs) that have recently been created. Most PPPs are based in the United States with the remainder located in Europe. "As a result, almost all monies raised by [PPPs] are channeled through first-world head offices, and any decisions made regarding how these are spent in developing countries are made by the CEOs, together with their senior staff and boards (statutory and advisory)" the overwhelming majority of whom are Caucasian, and are residents of either the United States or Europe. "Not one 'global' [PPP] is led by a person who is a developing-country national, and not one resides within one of the developing countries severely affected by neglected infectious diseases ... In addition, the main advisory boards of [PPPs] mostly have no people representing 'on-the-ground' communities in Africa, despite the fact that this input is critical if large studies are to have cultural sensitivity in resource-poor environments." [14] Recommendations have been made to at least four PPPs - International AIDS Vaccine Initiative, Multilateral Initiative on Malaria, Global Alliance for the Elimination of Lymphatic Filariasis and Medicines for Malaria Venture - to increase the diversity of representation on their boards so that they reflect the communities in which they are active [15].

It may sound trite, but priorities cannot just be set by donors (public or private) and the governments of developed and developing countries. All parties will need to do more to ensure that health care workers on the ground in developing countries and the people who will be the direct recipients of any new research initiatives are intimately involved in setting priorities for the research agenda. At a minimum this will require building up research capacity in the developing world, creating career structures for clinicians and scientists so that they are able to fully participate at the global level and developing initiatives to engage the communities that are affected by the diseases in question.

#### ii. Coordination of research efforts

Closely related to the question of priority setting is the coordination of research efforts undertaken by the various actors. This issue has recently been examined by Oxfam International in a briefing paper it produced on the crisis in health-related research and development in developing countries[16]. The report notes that within donor countries there is little coordination of activities and therefore governments do not necessarily know where they are allocating their research dollars. The result is that it is very unlikely that meaningful research targets can be established and even if they are that governments can be held accountable to these targets. What exists internally within countries is mirrored internationally with little coordination in the activities of different countries or between countries and other donors. This lack of coordination can lead to three types of problems: redundancy in the work that multiple countries or donors are undertaking, policy lacunae in the sense that there are essential tasks that are not being done and an incoherence in aims due to each actor having a different set of priorities and policies [17].

A social constructionist explanation for why some global health issues rise to prominence has to due with the effectiveness with which global health policy communities portray and communicate their "severity, neglect, tractability and benefit in ways that appeal to political leaders' social values and concepts of reality." In order to achieve issue ascendance and sustainability and therefore the necessary political attention, these portrayals also need to "be accompanied by institutions that create, negotiate, promote and sustain" them [18]. Poor coordination is one factor behind the failure to develop a strong policy community able to realize these objectives and the upshot is that certain health issues are overlooked in the allocation of R&D dollars despite the disease burden that they represent. The problem with coordination and health conditions that remain outside the spotlight may explain the priorities of the European Commission during its Sixth Research Framework Programme where 420 million Euros was spent on R&D for HIV and AIDS, tuberculosis, and malaria while all other neglected diseases combined received only about one-tenth of that amount [16].

To overcome the lack of coordination Oxfam is proposing the creation of a formalized framework to coordinate donor funding initiatives. Working within such a framework donors could examine the synergistic effects of different incentive mechanisms and at the same time help to ensure that money is allocated proportionately amongst the different competing priorities. A single agency could also keep records about which chemical compounds have already been rejected during screening, so that other researchers would not needlessly duplicate efforts. Finally, a formalized framework would provide "an avenue for recipient countries to participate and provide input about actual funding needs, priorities, and approaches that are appropriate in their countries." [16]

#### iii. Intellectual property rights and publicly funded research

Many of the drugs that eventually make it to the market are the product of basic research that has been publicly funded. According to a report commissioned for the United States (US) Senate, 15 of the 21 most important drugs introduced between 1965 and 1992 were developed using knowledge and techniques from federally funded research [19]. Much of this research is carried out in university settings. The passage of the *Bayh-Dole Act *in the US in 1980, and similar policies in other countries, gave universities the rights to any patents resulting from grants or contracts funded by any federal agency. Universities own patents on nearly 1 in 5 (19.2%) of the new molecular entities that received ''priority'' approval from the US Food and Drug Administration (FDA), including key patents on over one quarter of the HIV/AIDS drugs approved since 1988[20].

Once the legal and salary costs are considered (and all other costs are ignored) then a university technology transfer office with the median income, number of employees, and legal fees expended would generate an annual net income of only about US$30,000[21]. However, despite this marginal amount of money, many universities have restricted access to the products and knowledge of publicly funded research in the hopes of gaining revenue windfalls from exclusive licensing of patents to large commercial entities such as pharmaceutical companies. They hope to emulate Emory University which sold its rights to royalties for the antiretrovirals emtricitabine and tenofovir to Gilead Sciences and Royalty Pharma for a lump sum payment of US $525 million [22].

The issue of university-owned patents came to a head in 2001 when Médecins Sans Frontières (MSF) sought the permission of Yale University to use a generic version of stavudine, an antiretroviral drug, to treat South African patients. The university had granted an exclusive license for this product to Bristol-Myers Squibb. As a result of MSF's request and global attention, negotiations between Yale and Bristol-Myers Squibb lead the company to allow generic stavudine to be bought and sold within South Africa and to a 97% reduction in the price of the patented drug in South Africa[22].

Universities Allied for Essential Medicines (UAEM), a coalition of students and faculty at about 25 universities across North America, focuses on the role of universities as a starting point for closing the access and research gaps. UAEM has "put forward two specific policy proposals: (1) universities should adopt licensing provisions that facilitate access to their health-related innovations in poor countries, and (2) universities should promote research on neglected tropical diseases and find ways to work with nontraditional partners (such as developing-world research institutions and public-private partnerships) that seek to develop medicines for these diseases."[22]

#### iv. Intellectual property rights and patent thickets

Besides encouraging universities to acquire patents on their inventions the *Bayh-Dole Act*, along with the increasing commercialization of biomedical research, has lead to a proliferation of patents, especially in the field of biotechnology. The rapid increase in patents has the potential consequence of inhibiting research into diseases and treatments, a phenomenon that has acquired the name "the tragedy of the anticommons."[23] In this situation a resource, here biomedical knowledge, processes or products, may be underused because multiple owners each have a right to exclude others from a scarce resource and no one has an effective privilege of use. "Each upstream patent allows its owner to set up another tollbooth on the road to product development, adding to the cost and slowing the pace of downstream biomedical innovation."[23]

The emerging patent thicket represents a distinct threat to the traditions of open science required for progress. It has the potential to choke off collaboration while adding transaction costs to the price of doing research. It's impossible to quantify the damage done by this early stage scientific patenting because how can one count the number of researchers who abandon a line of research because they can't get access to the necessary licenses or execute the necessary material transfer agreements? How does one count the number of researchers who never even consider a line of research because someone else has already locked up the key patents?[24]

One way out of this dilemma is the creation of patent pools. The idea behind a patent pool is that multiple owners of patents dealing with the same subject matter are brought together by a single organization. These patents are then made available to interested parties -- researchers, manufacturers, etc. - on a non-exclusive basis subject to the payment of royalties. The advantage of patent pools is that it avoids negotiations with each individual patent holder[25].

In February 2009 GlaxoSmithKline (GSK) announced that it would put any chemicals and processes over which it controlled patent rights and that might contribute to research on neglected diseases into a patent pool in order to stimulate research[26]. GSK did not define what it meant by neglected diseases and since it does not consider HIV a neglected disease it has not included patents on these drugs in the pool it has created[27]. All the same, the move was welcomed by organizations such as Oxfam and MSF.

Patent pools are also being championed by UNITAID. The organization was set up to contribute to scaling up access to treatment for HIV/AIDS, malaria and tuberculosis, primarily for people in low-income countries, by leveraging price reductions for quality diagnostics and medicines and by accelerating the pace at which these are made available[28]. The UNITAID patent pool, once established, will be voluntary and it will function without any fundamental changes to the existing system of intellectual property rights for medicines. Brand-name companies may find the pool an attractive option as it will give them a very visible way of showing their commitment to access to medicines which could produce significant public relations benefits[29].

#### v. Clinical trial capacity

Clinical trials are essential in order to establish the efficacy and relative safety of new medicines. Especially in the case of Type III diseases, but also for Type I and II diseases, it is imperative that trials of new medicines be carried out in the developing countries where these diseases are most prevalent. However, despite the increase in the number of trials being undertaken in some developing countries [30] there are 3 structural barriers to expanding trial capacity, especially in sub-Saharan Africa, that need to be overcome: inadequate trial site capacity, limited regulatory ability to approve and supervise trials and the poor quality of ethical review committees[7].

According to the WHO Commission on Intellectual Property Rights (2006), as of about 2005 there were over 300 products for neglected diseases in development globally but the trial capacity in developing countries was unable to support this volume of potential products. WHO estimates that only 1 in 6 of its membership, primarily developed countries, have a well functioning regulatory system. Of the rest, one-third have either no system or one that barely functions and the remainder have systems at varying levels of development and operational capacity[31]. Ethics boards in US are much more likely to raise issues such as the need for local language consent forms, and confidentiality protection of participants than are boards in developing countries[32].

"Strengthening the R&D capacity in developing countries by investing in African owned health research centres capable of conducting clinical trials has thus been identified as an international priority to improve public health and, indirectly, development. Efforts should be focused on the establishment and strengthening of locally controlled and managed research centres able to pursue their own priorities and R&D agenda. The existence of internationally recognized institutes will also strengthen the position of African R&D priorities in international initiatives, and increases the ability to influence cash flows."[33] Addressing these issues and strengthening indigenous capacity will make it more likely that African (and other developing country) researchers will be able to set their own priorities and design their own protocols rather than relying on pharmaceutical companies and other organizations which may not fully understand the issues faced in developing countries.

Developing countries with some technological capacity have been relatively successful in building up an indigenous research capacity. Both Brazil and Cuba are producers of vaccines. Brazil is the world's largest producer and exporter of yellow fever vaccine and the meningitis B vaccine from Cuba was the first in the world against this strain of the bacteria[7]. In countries with a lesser degree of technological sophistication partnerships with Western organizations and countries are being developed and there have been some positive changes in funding directed at improving clinical trial capacity. The European and Developing Countries Clinical Trials Partnership focuses on the latter phases of clinical trials in sub-Saharan Africa for medicines, vaccines, and microbiocides against HIV/AIDS, malaria, and TB. The Fogarty Centre in the US provides research grants for collaborative research and capacity-building projects relevant to low- and middle-income nations[16].

### Proposals to expand research capacity

Governments, non-governmental organizations, intergovernmental organizations such as WHO, pharmaceutical companies and academics are all engaged in putting forward, and in some cases implementing, proposals for ways to overcome the problems with market failure that has so far characterized research into diseases of developing countries. This section looks at three proposals that have gained the most attention in recent years and examines their strengths and weaknesses. The first one is still at the stage of a proposal but the next two are being put into action.

#### i. Paying for innovation

Various authors have advanced schemes to promote research into neglected diseases that are based on paying the innovator from a prize fund[1,34-38]. One example of this type of scheme is the Health Impact Fund (HIF)[35]. Under this proposal companies could continue to market their products in the usual way under a patent or they could opt to register a new product (or a new use for an old product) with HIF. If the company chose this latter option then it would commit to sell the medicine wherever it is needed at the lowest feasible price based on production and distribution costs. Once the reward period ended the manufacturer would be required to offer free licenses to enable generic production. In return, it would be rewarded annually from the HIF's fixed pool of money on the basis of the product's global health impact in its first ten years following marketing approval, or on the assessed health impact of the new use of the product for a period of five years. In assessing the health impact, the HIF would "estimate the difference between (1) the actual health status of people who consumed the registered product and (2) the estimated health status of these people, had they not had access to the registered product or to any other products introduced less than two years before the registered product."[39] Financing for HIF would come from partner countries that commit to providing funding, currently pegged at about 0.03% of gross national income, for at least 12 years so that innovators would have some assurance about the payments they could expect to receive. HIF is estimated to cost about $6 billion annually.

Other proposals differ in major ways from HIF [34] but they all base their payments on the therapeutic value of the new products. This type of payment system potentially has significant advantages for companies since the greater the health benefits from their product(s) the more money they receive. Therefore, they are incentivized, at least in theory, to do research on diseases with major medical needs.

Making payments contingent on assessing therapeutic value presents its own set of problems the most prominent of which is where will the evidence come from to assess therapeutic value? Virtually all of the premarketing data on safety and efficacy comes from the pharmaceutical company that produces the medication and multiple studies have confirmed that this data is highly biased in favour of the company[40]. Furthermore, most of the clinical trials funded by pharmaceutical companies are short-term, often use surrogate endpoints and are placebo controlled[41]. If trials use an active comparator they are almost always non-inferiority trials meaning that the best that can be said is that two products are equivalent. Most pharmacoeconomic evaluations produced by manufacturers have significant methodological errors[42,43]. Relying on companies for postmarketing studies is also problematic. A review of 31 such projects in the United Kingdom concluded that company sponsored postmarketing surveillance studies had made only a limited contribution to the assessment of drug safety, principally because of weak study designs and difficulties in recruitment[44].

Aside from pharmaceutical companies there are limited resources available for generating information about therapeutic value. Public funding for clinical research is minimal even in developed countries and will be even scarcer in developing countries. Pogge and colleagues [39] recognize the problems with funding and propose that 10% of the HIF annual budget or $600 million be put aside to measure health impact. However, even with this money there are significant obstacles to gathering reliable data in developing countries including making accurate diagnoses, ensuring that the medicines are being used correctly, having the necessary technology to assess health status and accessing health outcomes in patients living in remote areas. Moreover, even if it is possible to assemble the necessary data it might take years to do so. Will companies be willing to wait that long before starting to receive payments from the prize fund? It is possible that payments could begin before all the necessary data is collected but this runs the risk of under or overestimating the therapeutic value of new products. Both possibilities would create financial problems for HIF. If therapeutic value was underestimated then additional money for these companies would need to be found from future HIF budgets decreasing the amount available for other products. If therapeutic value was overestimated, then although HIF could recover its excessive payments at a point in the future, it would still have restricted its payments in the present and have consequently paid less than what was appropriate for more valuable medications.

This list of difficulties is not to say that therapeutic value cannot be measured but before paying for innovation can be a reality there will have to be major strides in overcoming these problems. Clinical trial registries and registries for clinical trial results are one avenue for making information more public. There are also calls for safety and efficacy data filed with regulatory authorities for approval purposes to be made public [45]. However, both of these proposals still leave the generation of clinical information in the control of the pharmaceutical companies. One promising suggestion for overcoming this barrier is to separate the funding from the actual conduct of the clinical trials by requiring the companies to turn over the money for clinical trials to a neutral organization such as the National Institutes of Health. This organization would then choose the investigator(s) based on a peer reviewed process and would be responsible for controlling the data from the trials.

#### ii. Priority review vouchers

Beginning in September 2008, the US implemented a new system designed to increase research into neglected diseases. As described by Kesselheim [46] under this system the FDA will give a company marketing a treatment for a neglected disease in the US a "voucher" entitling it to a priority review for any other product it sells. The difference in time between a standard approval (12 months) and a priority review (6 months) could be worth as much as $300 million to a company depending on the sales potential of the product receiving the priority review. However, much of the research into neglected diseases is undertaken by small companies that are unlikely to have a large enough portfolio of products to benefit from a voucher. The legislation does allow vouchers to be sold but this type of deal making will lack transparency and may include intellectual property rights that will increase the cost and/or restrict the availability of products. According to Kesselheim [46] "the program reflects a growing trend in health policy toward reliance on substantial financial incentives to achieve a socially desirable outcome."

This is the only one of the 3 proposals that is not potentially subject to all 5 of the restrictions identified in the previous section. Because the vouchers are issued to a single company questions about priority setting and coordination of efforts do not apply. However, because research priorities are being left in the hands of a private for-profit entity there are serious doubts about whether such a system will have any effect in stimulating the necessary research.

The first of these vouchers was awarded in 2009 to Novartis upon FDA approval of its combination product (artemether/lumefantrine, trade name Coartem) for the treatment of malaria. "Yet artemether/lumefantrine was added to the WHO Essential Medicines List in 2002 and has been available in the developing world for years. There is therefore no benefit whatsoever to patients in the developing world from a US registration - and thus no reason to reward the company. Other companies could now come forward with even older drugs for tropical diseases that they never bothered to register in the US."[47] Furthermore, it is highly unlikely that the prospect of earning a voucher some years in the future will be enough of a stimulus to large pharmaceutical companies to initiate a program of research into neglected diseases. The value of a voucher will depend on the company having one or more "blockbuster" drugs ready for approval some years hence that could benefit from the voucher. Given the current track record of companies in developing blockbuster drugs having one that is approvable is hardly guaranteed. Small companies already engaged in research on neglected diseases that might be attracted by the possibility of a voucher may not be in a position to use it because they only have the resources to work on a few drugs at a time[46].

Finally, issuing priority reviews for drugs that are of a company's choosing distorts the intention of a priority review. This mechanism was intended to ensure that therapeutically valuable drugs would reach the market in an expedited manner. What the voucher system does is substitute therapeutic value for economic value. The two are often not synonymous. One proposal for reforming the voucher system is to auction off a fixed number of vouchers per year, and use the proceeds for a prize fund for neglected diseases[48]. However, this would just lead to the question of how to judge therapeutic value. On-the-whole, the wisest course would seem to be to abandon the voucher system in favour of some other method.

#### iii. Public-private partnerships

The definition of public-private partnerships (PPPs) is somewhat fluid. Kaplan defines them as "collaborative organizations between non-profit and for-profit organizations ... [that] are institutionalized with public intervention and/or funding"[49]. Moran et al [5] view "PPPs as public health driven not-for-profit organisations that drive neglected disease drug development in conjunction with industry groups." This definition includes groups that do *not *define themselves as PPPs. For instance, the Drugs for Neglected Diseases initiative (DNDi) sees itself as a primarily public group and the Institute for One World Health (iOWH) defines itself as a not-for-profit private company[5].

PPPs do not conduct drug development themselves. Their main functions are to integrate and co-ordinate multiple industry and academic partners and contractors along the drug development pipeline; allocate philanthropic and public funds to the 'right' kinds of R&D projects; and manage neglected disease R&D portfolios[5]. PPPs are the most advanced of the various alternatives to the usual method of researching and developing new drugs. A survey published in 2005 found that 47 of 63 new drugs for neglected diseases were being developed under the auspices of a PPP. One-third of these 47 drugs came out of PPPs involving large pharmaceutical companies and the balance were from PPPs working with a diversity of small firms, developing country firms, academics and the public sector[5].

The multinational companies that get involved in PPPs tend to focus their efforts on in-kind donations, for example, access to their molecular library or on early stage R&D leaving the costly development process to others. Some of these large companies also have an economic motivation for their involvement, as they see some possible commercial value in the form of spin-off research and obtaining exclusive rights to use the research in developed countries. On-the-other hand, small companies, biotechnology companies, contract research organizations and firms in developing countries have commercial imperatives different from those of large pharmaceutical companies. The economic position of these entities means that the smaller rewards that can come from involvement in a PPP may make commercial sense to them[7].

Although PPPs have enjoyed relative success there are three key problems that still need to be overcome in order to help ensure that they remain a viable option for enhancing research activity. The first one, the lack of representation from developing countries on the boards of PPPs, has already been discussed in the section on priority setting. Second is the question of intellectual property rights. Although some companies have opted to forgo patents on products that they are involved with, others still plan to apply for patents that may result in restricted access to knowledge and products. Whereas the Sanofi-Aventis-DNDi group decided not to file for a patent for its anti-malarial medicine, Novartis wants to obtain a patent for any products it develops through a PPP[50].

The third and most serious problem is the narrow funding base for PPPs. Direct R&D spending by PPPs between 2000 and 2006 is estimated to be just over $80 million with a similar amount from in-kind donations. Very little of that money has come from public sources with developed country governments contributing about 16% and the private sector has lagged even further behind with just 2% of research funding coming from that source. The bulk of the funding has originated from philanthropic organizations with almost 60% from the Gates Foundation alone[5]. Such heavy reliance on a single source means that the priorities of PPPs may be subject to the decisions of the board of the Gates Foundation.

Without an adequate guarantee of funding PPPs may have to make difficult decisions regarding the likelihood of success and possibly terminate projects prematurely. "A further constraint is the discrepancy between the long-term nature of the R&D process and the relatively short-term nature of funding pledges. Lack of certainty of continuing funding inhibits long-term planning by public-private partnerships. There may be a temptation to seek to do things more cheaply but not necessarily cost-effectively; promising research projects may be delayed and relationships with partners may be damaged because of a short-term approach."[7]

While this article does not allow an in-depth analysis of all of the suggestions for increasing PPP funding two mechanisms, one for increased public funding and one for private individual funding, will be described.

As a way to supplement the funding for PPPs, Moran [5] has proposed the creation of a publicly funded Industry R&D Facilitation Fund (IRFF) that would be a long-term grant fund of between US $130 million and US $190 million per year to underwrite industry participation in PPPs. IRFF funds would flow directly through to industry partners in the PPPs in order to pay them for neglected disease R&D, rather than financing PPPs as organizations and leaving it for the PPPs to distribute the money. The alleged benefits of this model are that "public funders can allocate resources across all PPP projects in the *exact *amounts needed *exactly when *they are needed, allowing all R&D projects to move forward simultaneously without delay" (emphasis in original), [5] although how such precision would be achieved is not described. Moran et al believe that the IRFF would remove the need for donors to choose among PPPs since all projects could be encompassed. This view seems somewhat naïve as it ignores the political decisions involved in choosing PPPs that fit with the priorities of different governments. The governing structure for the IRFF is not outlined and therefore it is not clear how the IRFF would ensure that politics does not become involved in decision making. Finally, given their history so far in funding PPPs, it is not clear why governments would now choose to commit to larger sums on an ongoing basis.

Ziemba, [51] in a paper produced for the WHO's Commission on Intellectual Property Rights, Innovation and Public Health, suggests that PPPs look at a funding mechanism, called "PharmaShares". Under this system when pharmaceutical stocks are sold or purchased the person doing the transaction is given the option to add a tax deductible amount for contribution to an independent "PharmaShares" fund. The fund then allocates money to PPPs that have applied to it. While this method may be useful in raising some money it hardly seems like a sustainable long-term option.

In summary, PPPs seem to be the most promising route for increasing R&D into neglected diseases but they continue to face significant barriers in accessing funding and that may ultimately limit how much they are able to contribute.

## Summary

There is actually a fair degree of unanimity amongst all of the actors involved in research for neglected diseases - they all agree that there is a pressing need for a major expansion in the level of R&D, that the present market based system cannot fulfill the task and that a new model is needed. Whatever that new model turns out to be, it will have to deal with the 5 barriers outlined in this paper. While each of them can be overcome the process of doing so will take time, commitment and cooperation among the public and private sectors, non-governmental organizations and the developing countries themselves. Finally, none of the three proposals considered here for expanding research is free from major limitations. The prize fund model has to deal with how to assess therapeutic value in a timely, independent and valid way, the voucher system is more a model of corporate welfare than it is a system for dealing with the needs of developing countries and PPPs face the challenge of finding a long-term stable source of financing. We are slowly moving ahead in expanding research capacity but unless the limitations of the prize fund model and PPPs are effectively dealt with we are also in danger of, if not taking a step backwards, then taking a step sideways rather than forward.

## Competing interests

The author declares that he has no competing interests.

## Pre-publication history

The pre-publication history for this paper can be accessed here:

http://www.biomedcentral.com/1472-698X/10/20/prepub
